# Association between COVID-19 and the Risk of Vascular Dementia: A Mendelian Randomisation Study of the Potential Cognitive Sequela of COVID-19

**DOI:** 10.3390/bs14060465

**Published:** 2024-05-30

**Authors:** Qing Han, Yue Ma, Wenting Ye

**Affiliations:** 1 Department of Social Policy and Intervention, University of Oxford, Oxford OX1 2ER, UK; 2 Faculty of Medicine, Macau University of Science and Technology, Macao, China; 3 School of Nursing, Southern Medical University, Guangzhou 510515, China; 4 Faculty of Psychology, Southwest University (SWU), Chongqing 400715, China

**Keywords:** COVID-19, vascular dementia, Mendelian Randomisation, cognition

## Abstract

A growing body of observational studies and Mendelian Randomisation analyses suggest an increased risk of Alzheimer’s disease and dementia following COVID-19 infection. However, evidence on the potential association between COVID-19 and vascular dementia, which is plausible given the vascular complications of COVID-19 infection, is still limited. In this study, we conducted a two-sample Mendelian Randomisation analysis to examine the potential causal relationship between COVID-19 phenotypes and the risk of vascular dementia, using summary data from large-scale GWASs. The two-sample Mendelian Randomisation analysis did not detect any significant associations of COVID-19 infection, COVID-19 hospitalisation, or critical COVID-19 with the risk of vascular dementia, with weighted average β values of −0.29 (95% CI: −0.84, 0.26; *p* = 0.301), −0.12 (95% CI: −0.36, 0.13; *p* = 0.345), and −0.07 (95% CI: −0.23, 0.09; *p* = 0.374), respectively. Our findings do not support the hypothesis that vascular dementia is one of the long-term sequelae of COVID-19.

## 1. Introduction

In May 2023, over three years after the start of the COVID-19 pandemic, the World Health Organization (WHO) declared that COVID-19 no longer constitutes a global health emergency. Following the end of the global pandemic status and the decrease in the virulence of circulating SARS-CoV-2 variants [1,2], the clinical and research focus has gradually been moved to the long-term sequelae of COVID-19 [3]. An extensive body of literature has shown that COVID-19, especially during the pre-Omicron period, can have long-term consequences for multiple systems of the human body, including the neurological and cognitive systems [3]. A systematic review and meta-analysis at the early pandemic stage showed that there were over 50 persistent symptoms of COVID-19 between 14 and 110 days post-infection [4]. Our previous meta-analysis of studies with at least a one-year follow-up of COVID-19 patients also found a high prevalence of long-term symptoms, with the most common being fatigue, respiratory symptoms, mental health, and cognitive symptoms (including memory loss/memory complaints, concentration difficulties, and insomnia) [5].

In fact, a growing body of observational studies [6,7,8] and Mendelian Randomisation (MR) analyses [9,10,11] suggests an increased risk of cognitive decline and dementia incidence following SARS-CoV-2 infection or severe COVID-19. However, the underlying mechanisms linking COVID-19 and dementia are still a mystery, with the main paradox being that COVID-19 is an infectious respiratory disease whereas dementia is a chronic progressive neurodegenerative disorder [12]. From a mechanistic and pathological perspective, among the different types of late-onset dementia, the association between vascular dementia and COVID-19 is more plausible given the vascular complications of COVID-19 infection [13]. Nevertheless, previous studies on this topic have been mainly focused on Alzheimer’s disease or overall dementia, and there is still a lack of evidence on the link between COVID-19 and the risk of vascular dementia.

In this study, we examined the potential causal links between COVID-19 phenotypes and vascular dementia using Mendelian Randomisation analyses, a causal inference technique through genetic instrumental variables [14]. Based on summary data from large-scale genome-wide association studies (GWASs), the MR analysis used single-nucleotide polymorphisms (SNPs) associated with exposure as the instrumental variables for exposure, thus providing an approach to assess the causal link between exposure and outcome variables while avoiding the bias from unmeasured confounding variables and reverse causation [14,15]. In fact, a common limitation of observational studies on COVID-19 and dementia is the limited follow-up time between COVID-19 infection and the development or diagnosis of dementia, which makes the temporal sequence hard to determine. In MR analysis, this issue can be resolved through the nature of the study design [14,15], where the instrumental variants of COVID-19 occur before the development of dementia.

Valid causal estimates between exposure and outcome variables can be obtained from MR analysis if the following three assumptions are met: (1) Relevance assumption, which means that the instrumental SNPs are associated with the exposure variable (i.e., COVID-19 phenotypes). (2) Independence assumption, which means that there are no unmeasured confounders (or common causes) of instrumental SNPs and the outcome variable (i.e., vascular dementia), and that the instrumental SNPs are not associated with confounders for the exposure–outcome association. (3) Exclusion restriction assumption, which means that the instrumental SNPs affect the outcome only through their effect on the exposure (i.e., no horizontal pleiotropy).

## 2. Materials and Methods

### 2.1. Data Sources and Phenotype Definitions

#### 2.1.1. GWAS Data for Exposure Variables—COVID-19 Phenotypes

**Figure 1:**
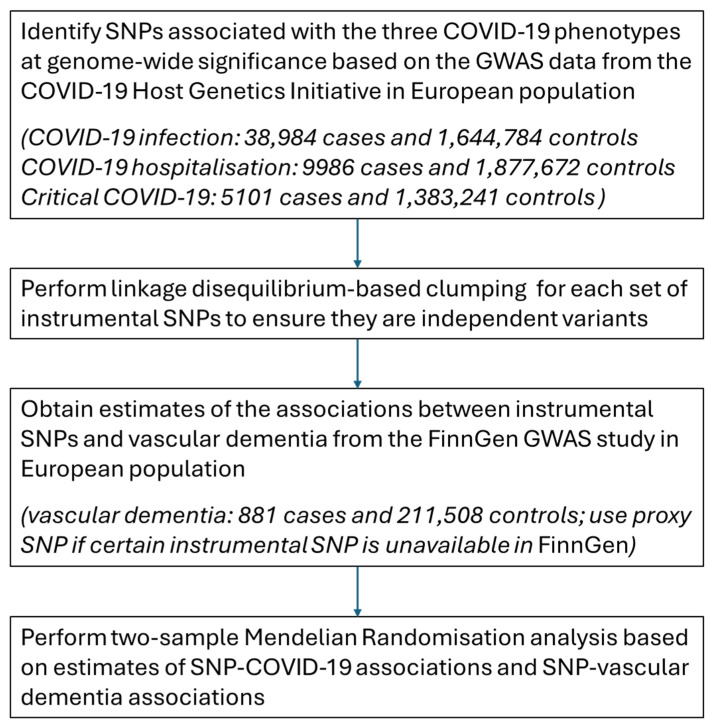
Study flow chart of data collection and preparation for the two-sample Mendelian Randomisation analysis on COVID-19 and the risk of vascular dementia. Note: SNP = single-nucleotide polymorphisms; GWAS = genome-wide association study.

We presented the study flow chart of data collection and preparation in Figure 1. We obtained GWAS summary-level data (European population) of three COVID-19 phenotypes (COVID-19 infection, COVID-19 hospitalisation, critical COVID-19) from the COVID-19 Host Genetics Initiative (version R5) [16]. The COVID-19 Host Genetics Initiative (https://www.covid19hg.org/, accessed on 12 April 2024) [16] is an international collaboration aimed at exploring genetic determinants of the susceptibility to and severity of COVID-19, and included multiple COVID-19 genetic studies for the genome-wide association meta-analyses. A total of 44 studies were included in the GWAS meta-analysis of COVID-19 infection, 29 studies for COVID-19 hospitalisation and 16 studies for critical COVID-19 [17]. Each eligible individual study was required to have at least 50 cases for specific analysis, though the sample representativeness could not be guaranteed. In the current study, we used GWAS data from populations of European ancestry.

In detail, the GWAS data for COVID-19 infection were based on genetic data from 38,984 COVID-19 cases (including laboratory-confirmed, clinical-confirmed, and self-reported SARS-CoV-2 infection) and 1,644,784 controls without recorded or reported SARS-CoV-2 infection; the GWAS data for COVID-19 hospitalisation were based on genetic data from 9986 cases who were admitted to hospital due to COVID-19 and 1,877,672 controls without COVID-19; and the GWAS data for critical COVID-19 were based on genetic data from 5101 cases who were hospitalised due to COVID-19 and required respiratory support or whose causes of death were associated with COVID-19 and 1,383,241 controls without COVID-19 [17].

#### 2.1.2. GWAS Data for the Outcome Variable—Vascular Dementia

We obtained GWAS summary-level data (European population) of diagnosed vascular dementia from the FinnGen research project (https://www.finngen.fi/en, accessed on 12 April 2024) [18], which holds genome and health data from Finnish biobank donors with the aim of understanding the genetic basis of diseases [18]. The FinnGen sample was not epidemiologically representative due to the recruitment strategy; all participants included in the GWAS analysis were of European ancestry (more specifically, Finnish European).

The GWAS data for vascular dementia in FinnGen were based on 881 cases who were diagnosed with vascular dementia according to the ICD-10 codes (i.e., International Classification of Diseases 10th Revision) in their health records and 211,508 controls without a diagnosis of vascular dementia [18].

### 2.2. Selection Procedure of Genetic Instruments

#### 2.2.1. Identification of SNPs Associated with COVID-19 Phenotypes

According to the COVID-19 Host Genetics Initiative GWAS data, SNPs associated with each of the three COVID-19 phenotypes at genome-wide significance (i.e., *p* < 5 × 10^−8^) were used as genetic instruments for that corresponding phenotype.

#### 2.2.2. SNP Clumping

Then, linkage-disequilibrium-based SNP clumping was performed, with the criterion of r^2^ < 0.001 and the clumping distance of 100 kb, to ensure that the instrumental SNPs for each COVID-19 phenotype were independent variants.

#### 2.2.3. Proxy SNPs (If Needed)

If the data of an instrumental SNP for COVID-19 were not available in the FinnGen GWAS of vascular dementia, we searched for another SNP available in FinnGen that was in strong linkage disequilibrium with that instrumental SNP (with the criterion of r^2^ > 0.80) and used it instead in the following analyses.

### 2.3. Two-Sample Mendelian Randomisation Analysis

**Figure 2:**
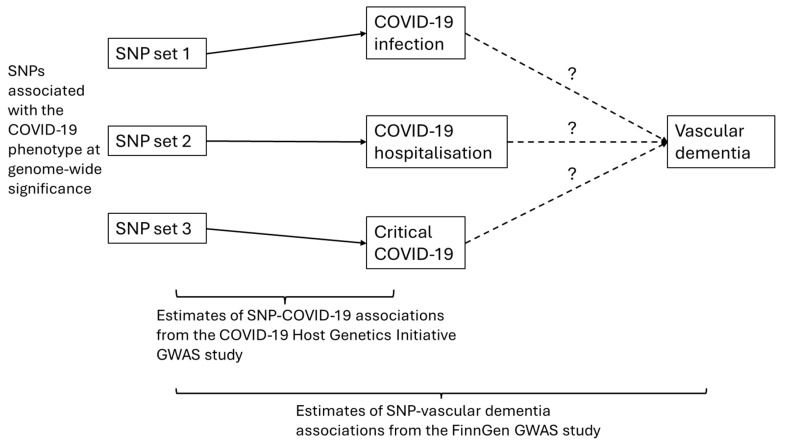
Graphical illustration of the two-sample Mendelian Randomisation analysis. Note: SNP = single-nucleotide polymorphisms; GWAS = genome-wide association study. “?” refers to the associations to be assessed.

Figure 2 shows the design and illustration of the two-sample Mendelian Randomisation analysis. The COVID-19-associated SNPs were used as genetic instruments for the causal inference between COVID-19 phenotypes and vascular dementia. Taking COVID-19 infection as an example, if there was a causal effect from COVID-19 infection to vascular dementia (i.e., COVID-19 infection increased or decreased the risk of developing vascular dementia), the SNPs associated with COVID-19 infection would also be expected to have an association with vascular dementia (i.e., vertical pleiotropy). The estimate of this causal effect was calculated based on the associations between those instrumental SNPs and COVID-19 infection (obtained from the COVID-19 Host Genetics Initiative GWAS summary-level data) and the associations between those instrumental SNPs and vascular dementia (obtained from the FinnGen GWAS summary-level data).

For each COVID-19-associated instrumental SNP, a Wald ratio was calculated, which is a ratio of the β coefficient of the SNP–vascular dementia association (i.e., log (odds ratio)) to the β coefficient of the SNP–COVID-19 phenotype association. The Wald ratio is an estimate of the causal effect of the COVID-19 phenotype on the risk of vascular dementia. When there was more than one instrumental SNP for a COVID-19 phenotype, which was a common situation, we used the inverse variance weighted method to combine the causal estimates calculated based on those individual SNPs, and obtained a final causal estimate. The Q test was used to assess the heterogeneity across the multiple causal estimates calculated based on the individual SNPs.

### 2.4. Sensitivity Analyses

To account for the potential violation of Mendelian Randomisation assumptions, we conducted the following sensitivity analyses: (1) We used funnel plot and MR-Egger regression to assess the possibility of horizontal pleiotropy, which means that the SNP can influence vascular dementia through a pathway other than COVID-19 and could bias the estimates from Mendelian Randomisation analysis [19]. (2) Instead of the inverse variance weighted method, we used multiple alternative methods to combine the causal estimates from individual SNPs, including the maximum likelihood method, MR-Egger regression, the weighted median method, and the weighted mode method. (3) We conducted a leave-one-out sensitivity analysis to check if the combined causal estimate was disproportionately driven by a single SNP.

The two-sample Mendelian Randomisation analyses were conducted using R packages TwoSampleMR and MRInstruments and the MR-Base platform (www.mrbase.org, accessed on 12 April 2024) [15].

## 3. Results

### 3.1. Eligible Genetic Instruments

Following the selection procedure of genetic instruments, we included the following genetic instruments in our MR analyses based on their signals in the COVID-19 Host Genetics Initiative GWAS [16,17] and their availability status in the FinnGen GWAS [18]: seven instrumental SNPs for COVID-19 infection (rs10936744, rs12482060, rs17078348, rs2271616, rs4971066, rs643434, and rs757405), six instrumental SNPs for COVID-19 hospitalisation (rs13050728, rs2109069, rs2660, rs35081325, rs505922, and rs622568), and nine SNPs for critical illness of COVID-19 (rs10860891, rs111837807, rs13050728, rs2109069, rs2237698, rs2384074, rs35081325, rs622568, and rs77534576). These instrumental genetic variants were associated with COVID-19 phenotypes mainly through biological mechanisms related to lung function or autoimmune and inflammatory diseases [17]. The variances explained by the instrumental SNPs for the exposure traits (i.e., COVID-19 phenotypes) were low to moderate, as assessed in previous MR studies using the same GWAS data source [9,10].

### 3.2. Results of Two-Sample Mendelian Randomisation Analyses

#### 3.2.1. COVID-19 Infection and the Risk of Vascular Dementia

**Figure 3:**
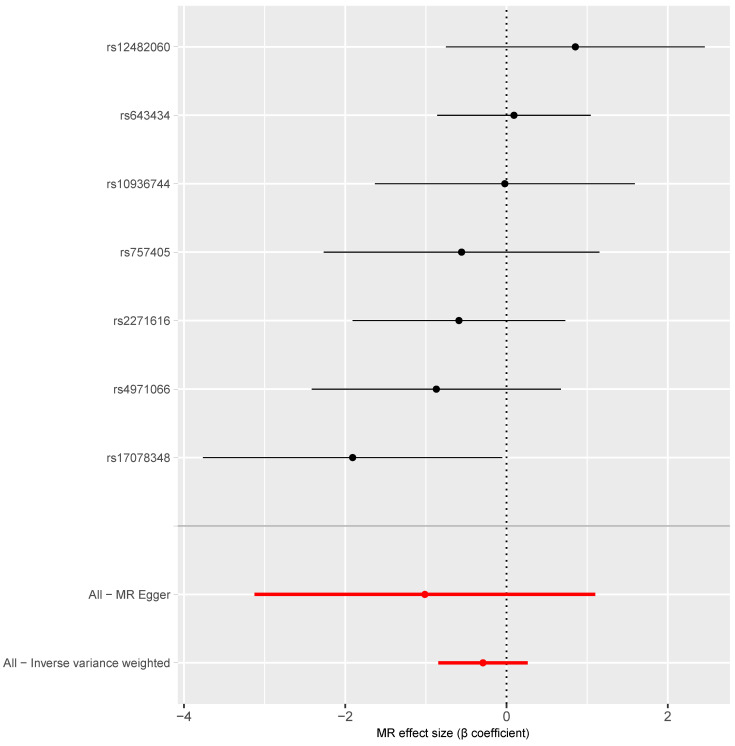
Forest plot of the MR estimates of the association between COVID-19 infection and the risk of vascular dementia. Note: The black dots and lines refer to the estimates and 95% confidence intervals based on individual SNPs; the red dots and lines refer to the pooled estimates and 95% confidence intervals.

As shown in Figure 3, the causal estimates for the association between COVID-19 infection and the risk of vascular dementia based on each of the seven instrumental SNPs ranged from −1.91 to 0.85 (all *p* for these β coefficients > 0.05). There was no substantial heterogeneity across the causal estimates calculated based on the seven individual SNPs (Q = 6.44, *p* = 0.376). After combining these estimates using the inverse variance weighted method, the weighted average causal estimate did not reach statistical significance (β = −0.29, 95% CI: −0.84, 0.26, *p* = 0.301; Table 1, Figure 3).

#### 3.2.2. COVID-19 Hospitalisation and the Risk of Vascular Dementia

**Figure 4:**
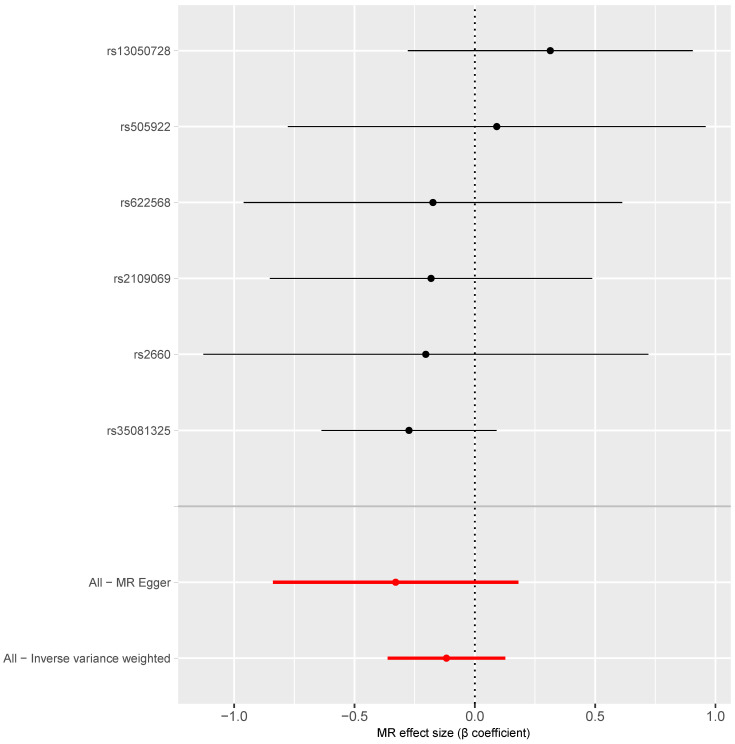
Forest plot of the MR estimates of the association between COVID-19 hospitalisation and the risk of vascular dementia. Note: The black dots and lines refer to the estimates and 95% confidence intervals based on individual SNPs; the red dots and lines refer to the pooled estimates and 95% confidence intervals.

Figure 4 shows that the causal estimates for the association between COVID-19 hospitalisation and the risk of vascular dementia based on each of the six instrumental SNPs ranged from −0.27 to 0.31 (all *p* for these β coefficients > 0.05). There was no substantial heterogeneity across the causal estimates calculated based on the six individual SNPs (Q = 3.06, *p* = 0.691). The inverse variance weighted average causal estimate showed no significant association between COVID-19 hospitalisation and the risk of vascular dementia (β = −0.12, 95% CI: −0.36, 0.13, *p* = 0.345; Table 1, Figure 4).

#### 3.2.3. Critical COVID-19 and the Risk of Vascular Dementia

**Figure 5:**
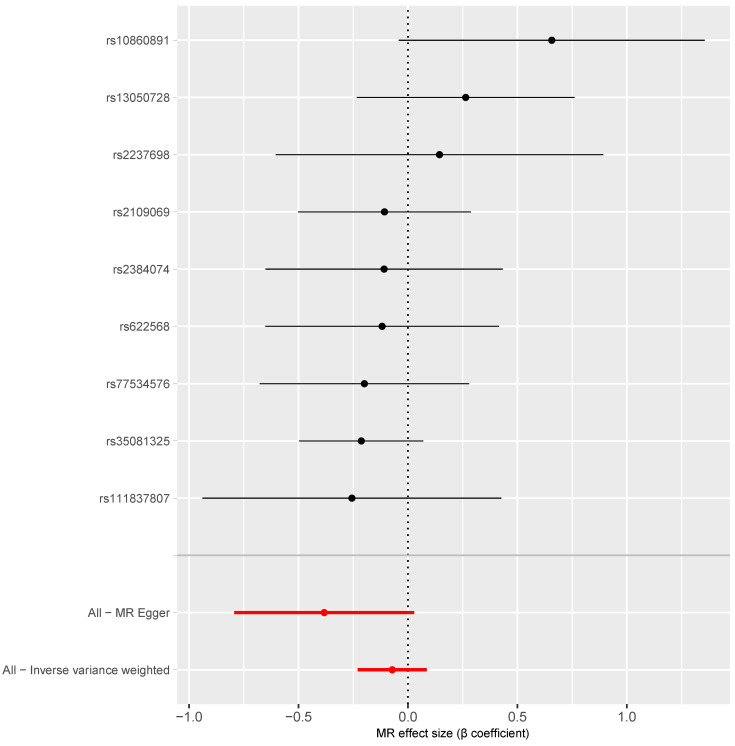
Forest plot of the MR estimates of the association between critical COVID-19 and the risk of vascular dementia. Note: The black dots and lines refer to the estimates and 95% confidence intervals based on individual SNPs; the red dots and lines refer to the pooled estimates and 95% confidence intervals.

Figure 5 shows that the causal estimates for the association between critical COVID-19 and the risk of vascular dementia based on each of the nine instrumental SNPs ranged from −0.26 to 0.66 (all *p* for these β coefficients > 0.05). There was no substantial heterogeneity across the causal estimates calculated based on the nine individual SNPs (Q = 7.83, *p* = 0.450). The inverse variance weighted average causal estimate showed no significant association between critical COVID-19 and the risk of vascular dementia (β = −0.07, 95% CI: −0.23, 0.09, *p* = 0.374; Table 1, Figure 5).

### 3.3. Results of Sensitivity Analyses

#### 3.3.1. COVID-19 Infection and the Risk of Vascular Dementia

**Figure 6:**
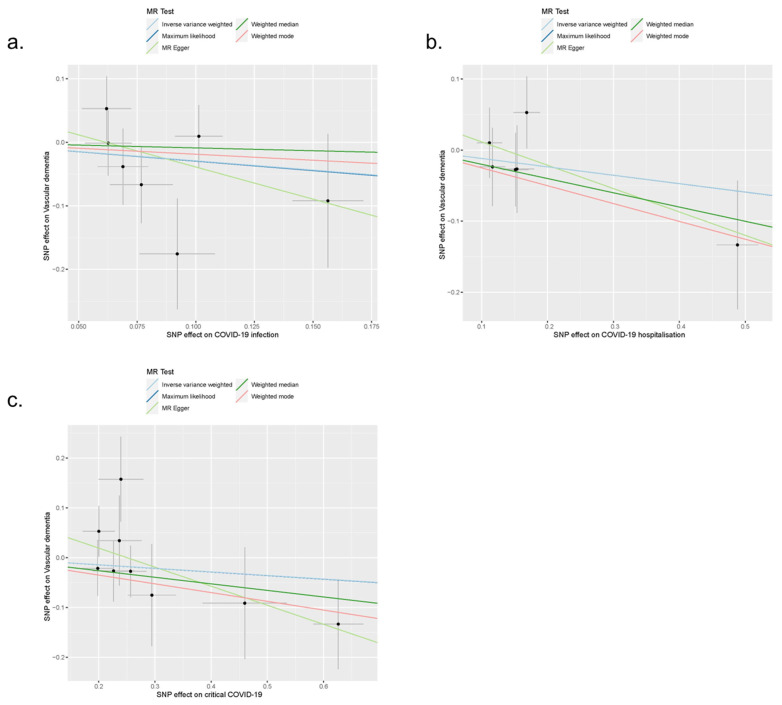
Scatter plots of the MR estimates between COVID-19 infection (**a**), COVID-19 hospitalisation (**b**), and critical COVID-19 (**c**) and the risk of vascular dementia. Note: The light blue and dark blue lines are overlapped due to identical estimates from the inverse variance weighted method and the maximum likelihood method.

Horizontal pleiotropy is a violation of the Mendelian Randomisation assumptions. In this analysis, the intercept of MR-Egger regression, a measure of the magnitude of horizontal pleiotropy, was 0.062 (standard error, SE = 0.090; *p* = 0.518), which suggested no evidence of directional horizontal pleiotropy (Figure 6). The funnel plot of the seven SNP-specific causal estimates also showed no clear sign of directional horizontal pleiotropy (Supplementary Figure S1).

The results of the sensitivity analyses using the maximum likelihood method, MR-Egger regression, weighted median method, and weighted mode method to combine the causal estimates from individual SNPs also showed no significant causal association between COVID-19 infection and the risk of vascular dementia, with the β coefficients ranging from −1.01 to −0.09 (all *p* > 0.05; Figure 6, Table 1). The leave-one-out sensitivity analysis did not reveal any strong influential SNPs in this MR analysis (Supplementary Figure S2).

#### 3.3.2. COVID-19 Hospitalisation and the Risk of Vascular Dementia

In this MR analysis, both the test of the intercept of MR-Egger regression (intercept = 0.044, SE = 0.048, *p* = 0.408) and the funnel plot of the six SNP-specific causal estimates suggested there was no evidence of directional horizontal pleiotropy (Figure 6, Supplementary Figure S3).

The sensitivity analyses using the maximum likelihood method, MR-Egger regression, weighted median method, and weighted mode method also showed no significant causal association between COVID-19 hospitalisation and the risk of vascular dementia, with the β coefficients ranging from −0.33 to −0.12 (all *p* > 0.05; Figure 6, Table 1). The leave-one-out sensitivity analysis did not reveal any strong influential SNPs in this MR analysis (Supplementary Figure S4).

#### 3.3.3. Critical COVID-19 and the Risk of Vascular Dementia

In this MR analysis, the funnel plot of the nine SNP-specific causal estimates showed slight asymmetry (Supplementary Figure S5), but the test of the intercept of MR-Egger regression indicated no evidence of directional horizontal pleiotropy (intercept = 0.096, SE = 0.060, *p* = 0.153; Figure 6).

The sensitivity analyses using the maximum likelihood method, MR-Egger regression, weighted median method, and weighted mode method also showed no significant causal association between critical COVID-19 and the risk of vascular dementia, with the β coefficients ranging from −0.38 to −0.07 (all *p* > 0.05; Figure 6, Table 1). The leave-one-out sensitivity analysis did not reveal any strong influential SNPs (Supplementary Figure S6).

## 4. Discussion

In this study, we evaluated the potential causal effects of COVID-19 infection, COVID-19 hospitalisation, and critical COVID-19 on the risk of developing vascular dementia using the two-sample Mendelian Randomisation analyses [14,19] based on the summary-level data of large-scale GWASs [16,18]. However, the MR analyses did not detect any significant causal associations between the COVID-19 phenotypes and vascular dementia risk, and our sensitivity analyses revealed no clear violation of MR assumptions or other possible biases in the estimates.

According to the previous literature on COVID-19 and dementia, multiple observational studies [6,7,8] and Mendelian Randomisation studies [9,10,11] have indicated a positive association (i.e., an increased risk of dementia following infection or severe illness with COVID-19), though the majority of those studies have focused on the risk of Alzheimer’s disease or overall dementia. Regarding the evidence from observational studies, a large-scale cohort study in the United States based on electronic health records in the UnitedHealth Group Clinical Research Database showed that, after at least four months following COVID-19 infection, COVID-19 patients had a significantly increased incidence of memory difficulties and dementia compared with those who did not have COVID-19 [7]. In addition, the study showed that compared with those with viral lower respiratory tract illness, COVID-19 patients still had an increased risk of developing dementia [7]. Another large-scale cohort study based on the TriNetX electronic health records network showed that, compared with patients with any other respiratory infections, COVID-19 patients had an increased risk of dementia persistently throughout the 2-year observation period [6]. A data-driven analysis using electronic health records from the RECOVER initiative indicated that the dementia incidence was significantly higher in patients 30–180 days after COVID-19 infection compared to non-infected patients [8].

As for the previous evidence from Mendelian Randomisation studies, one study suggested that COVID-19 hospitalisation and critical COVID-19 had a significant causal effect on the increased risk of Alzheimer’s disease, but no causal effect of general COVID-19 infection on Alzheimer’s disease was detected [20]. Consistently, an updated MR study also found that COVID-19 hospitalisation and critical COVID-19, but not general COVID-19 infection, were causally associated with the increased risk of Alzheimer’s disease [11]. In contrast, an MR study on cognitive performance suggested that COVID-19 infection, but not COVID-19 hospitalisation or critical COVID-19, had a significant causal effect on lower cognitive performance [10]. Another MR study showed that COVID-19 hospitalisation was significantly associated with a higher risk of Alzheimer’s disease, while the phenotypes of COVID-19 infection and critical COVID-19 were nominally associated with a higher risk of Alzheimer’s disease [9]; that study also conducted MR analyses on frontotemporal dementia and Lewy body dementia, but found no signs of causal links between COVID-19 phenotypes and these two dementia subtypes [9].

Our MR analyses filled the research gap of the lack of causal inference studies on COVID-19 and vascular dementia and also suggested that the observed associations between COVID-19 and increased risk of dementia in previous studies were not likely to be explained by vascular mechanisms [3]. Except for the hypothesised vascular pathway (i.e., COVID-19 leads to endothelial dysfunction and thrombosis [13] in cerebral vessels and thus increases the risk of vascular dementia or Alzheimer’s disease), there are other potential mechanisms that may link COVID-19 to an increased dementia risk, such as inflammatory or immune-mediated pathways [3,21]. As shown in a clinical study, the oxidative stress and inflammatory markers were significantly increased in the brains of COVID-19 patients compared with controls [22]. Another study based on brain imaging of UK Biobank participants found a reduced grey matter thickness and overall brain size in COVID-19 patients compared with controls [23].

Our results differed from those in previous observational and MR studies because previous studies used Alzheimer’s disease or overall dementia as the outcome variable (instead of vascular dementia). Therefore, our observation could reflect the heterogeneity between vascular dementia and Alzheimer’s disease. Future observational studies with vascular dementia as the outcome, and research on relevant mechanisms is needed. On the other hand, the negative findings could also be due to the weak instrument bias, which refers to the bias due to the weak association between instrumental SNPs and COVID-19 phenotypes. Nevertheless, previous MR studies using similar instrumental SNPs (obtained from the same data source: the COVID-19 Host Genetics Initiative) detected significant associations with Alzheimer’s disease [9,10]. In addition, Tang et al. [10] assessed the independence assumption and the exclusion restriction assumption by searching for significant associations between these instrumental SNPs and potential confounders or mediators (e.g., education level and body mass index) across previous GWASs using PhenoScanner [24]; no clear violation of the assumptions was found. Our sensitivity analyses also revealed no sign of horizontal pleiotropy.

There are several limitations that should be considered when interpreting our results. Firstly, the reliability of our MR estimates relies on the availability and quality of data from the GWASs. In our analysis, the number of patients (or cases) in the GWAS for vascular dementia was relatively low; future GWASs with larger numbers of vascular dementia patients can provide more ideal data sources for the MR analyses. Secondly, the clinical classification of COVID-19 phenotypes (e.g., distinguishing critical and non-critical COVID-19) and vascular dementia could sometimes be difficult. Nevertheless, both GWASs defined these phenotypes based on consensus guidelines or specific clinical diagnosis codes, which increased the reliability of the classification between cases and controls. Thirdly, since all GWAS data used in our study were based on participants of European ancestry, the generalisability of our findings to other ethnic groups remains unclear. Finally, in our analysis, the GWAS data for COVID-19 phenotypes were obtained before the circulation of Omicron variants. As shown in a large-scale cohort study, there was an increased risk of cognition or memory disorders in both Omicron-infected patients and Delta-infected patients compared with non-COVID-19 individuals, but the magnitude of the risk increase was smaller in Omicron-infected patients [25]. Therefore, future studies on the cognitive sequelae of Omicron variants are needed.

## 5. Conclusions

In conclusion, our Mendelian Randomisation study did not find any evidence of a causal relationship between COVID-19 infection, COVID-19 hospitalisation, or critical COVID-19 and the risk of vascular dementia, and thus it does not support the hypothesised vascular pathways linking COVID-19 and dementia. COVID-19 is a complex disease with multisystem manifestations and long-term sequelae [26]; whether the observed association between COVID-19 and dementia in previous studies is causal, and if yes, the underlying mechanisms linking these two complex diseases, still warrant further research.

## Figures and Tables

**Table 1:** Summary results of the MR estimates of the associations between COVID-19 phenotypes and the risk of vascular dementia.

COVID-19 Phenotype	Method	Number of SNPs	β	SE	*p*
COVID-19 infection	Inverse variance weighted (main analysis)	7	−0.29	0.28	0.302
	Maximum likelihood	7	−0.30	0.28	0.281
	MR Egger	7	−1.01	1.08	0.391
	Weighted median	7	−0.09	0.35	0.800
	Weighted mode	7	−0.19	0.43	0.678
COVID-19 hospitalisation	Inverse variance weighted (main analysis)	6	−0.12	0.12	0.345
	Maximum likelihood	6	−0.12	0.13	0.346
	MR Egger	6	−0.33	0.26	0.275
	Weighted median	6	−0.20	0.15	0.179
	Weighted mode	6	−0.25	0.17	0.197
Critical COVID-19	Inverse variance weighted (main analysis)	9	−0.07	0.08	0.374
	Maximum likelihood	9	−0.07	0.08	0.373
	MR Egger	9	−0.38	0.21	0.111
	Weighted median	9	−0.13	0.10	0.205
	Weighted mode	9	−0.18	0.11	0.155

Note: MR = Mendelian Randomisation; SNP = single-nucleotide polymorphism; SE = standard error.

## Data Availability

The original contributions presented in this study are included in the article/Supplementary Materials, and further inquiries can be directed to the corresponding author.

## Supplementary Materials

The following supporting information can be downloaded at https://www.mdpi.com/article/10.3390/bs14060465/s1, Figure S1: Funnel plot of MR estimates of COVID-19 infection and the risk of vascular dementia; Figure S2: Results of leave-one-out sensitivity analysis of COVID-19 infection and the risk of vascular dementia; Figure S3: Funnel plot of MR estimates of COVID-19 hospitalisation and the risk of vascular dementia; Figure S4: Results of leave-one-out sensitivity analysis of COVID-19 hospitalisation and the risk of vascular dementia; Figure S5: Funnel plot of MR estimates of critical COVID-19 and the risk of vascular dementia; Figure S6: Results of leave-one-out sensitivity analysis of critical COVID-19 and the risk of vascular dementia.
