# Supplementary material for: Association between COVID-19 and the Risk of Vascular Dementia: A Mendelian Randomisation Study of the Potential Cognitive Sequela of COVID-19
Source: Behav Sci (Basel). 2024 May 30;14(6):465. doi: 10.3390/bs14060465 (PMC11200909; doi:10.3390/bs14060465)
Supplement: Supplementary file 1 [file behavsci-14-00465-s001.zip › behavsci-2986373-supplementary.pdf]

Supplementary Figures

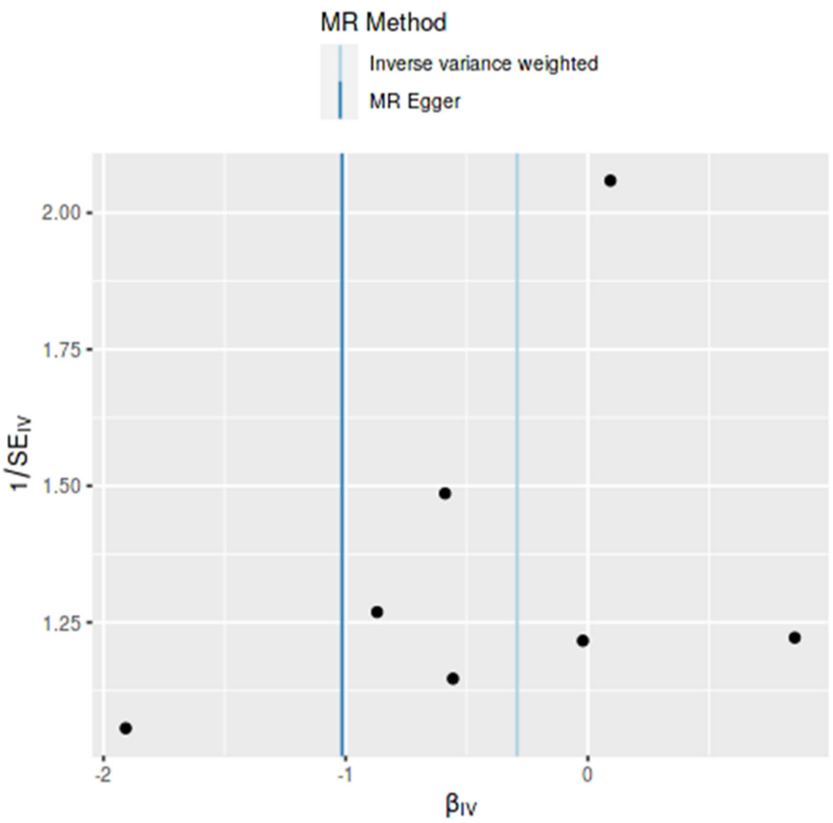

Supplementary Figure S1. Funnel plot of MR estimates of COVID-19 infection and the risk of vascular dementia

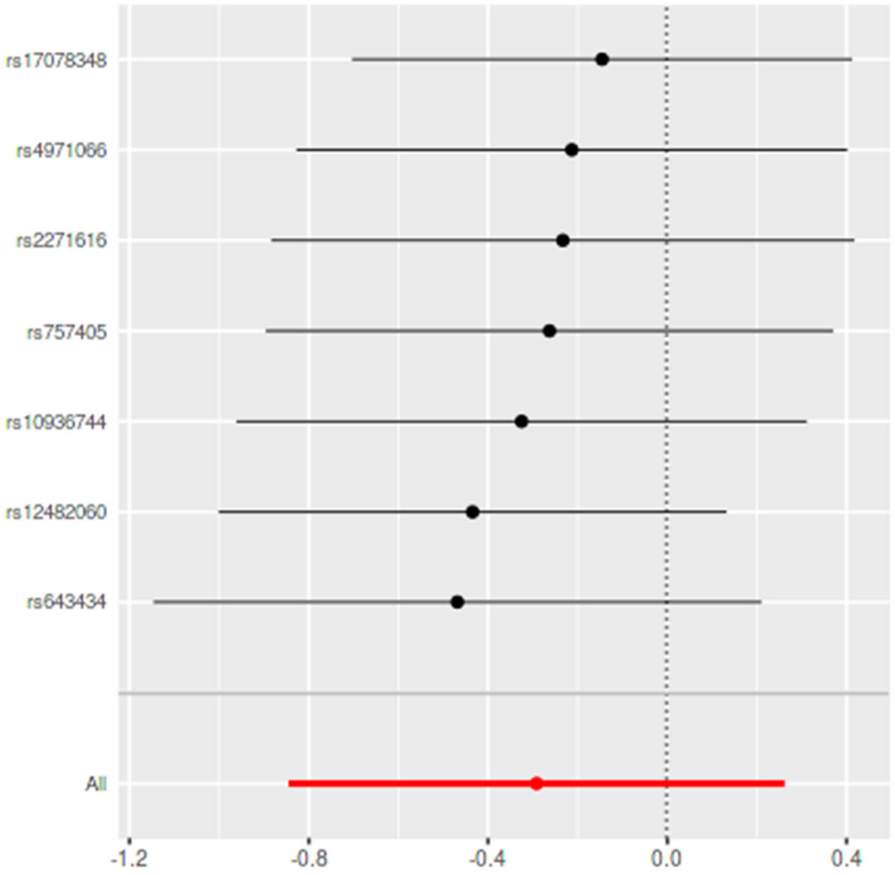

Supplementary Figure S2. Results of leave-one-out sensitivity analysis of COVID-19 infection and the risk of vascular dementia

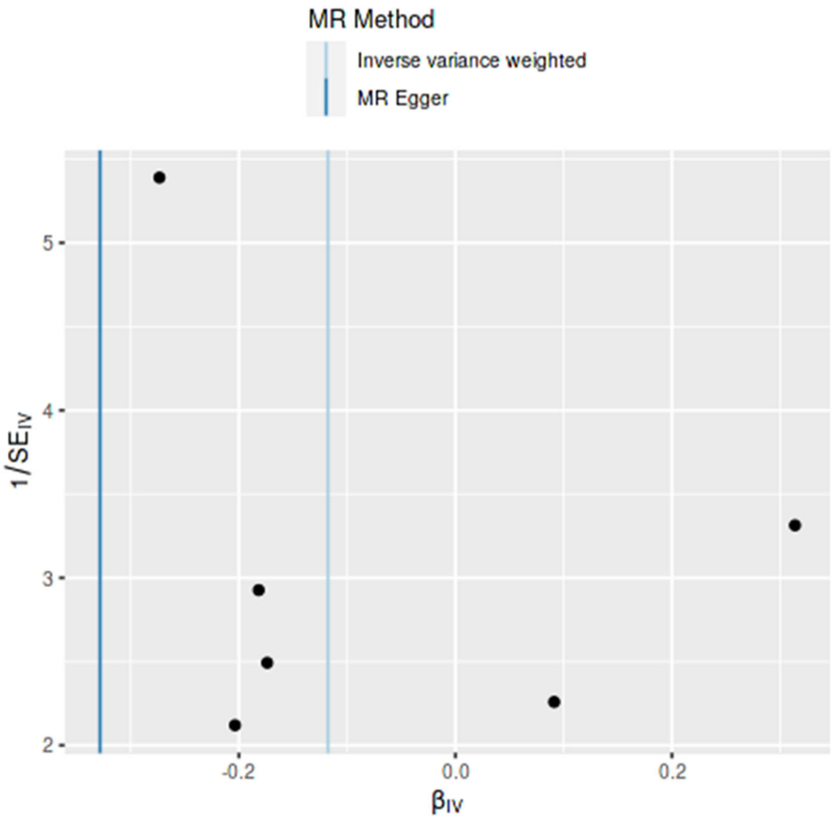

Supplementary Figure S3. Funnel plot of MR estimates of COVID-19 hospitalisation and the risk of vascular dementia

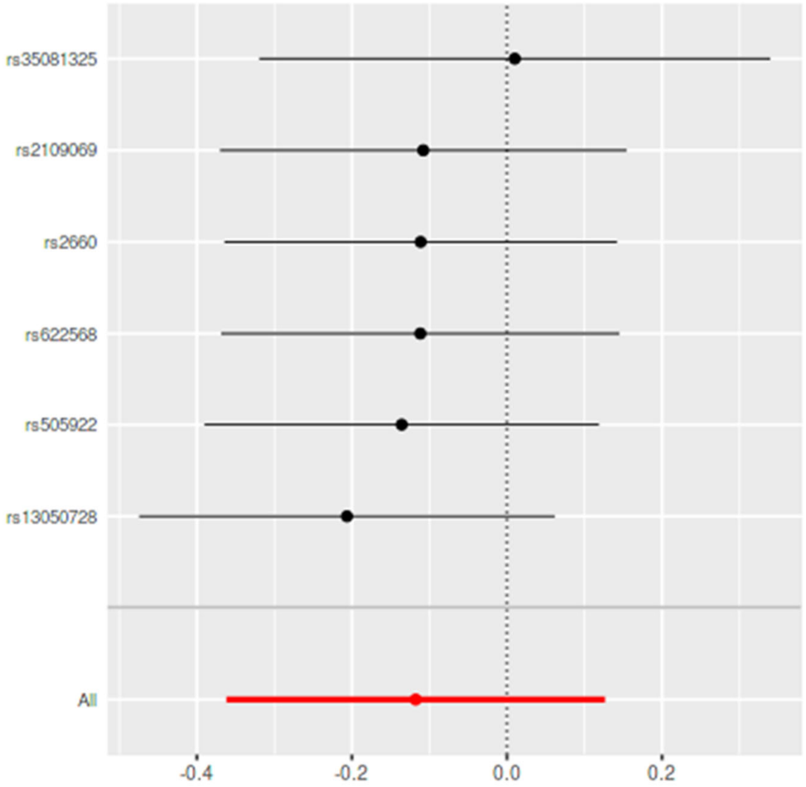

Supplementary Figure S4. Results of leave-one-out sensitivity analysis of COVID-19 hospitalisation and the risk of vascular dementia

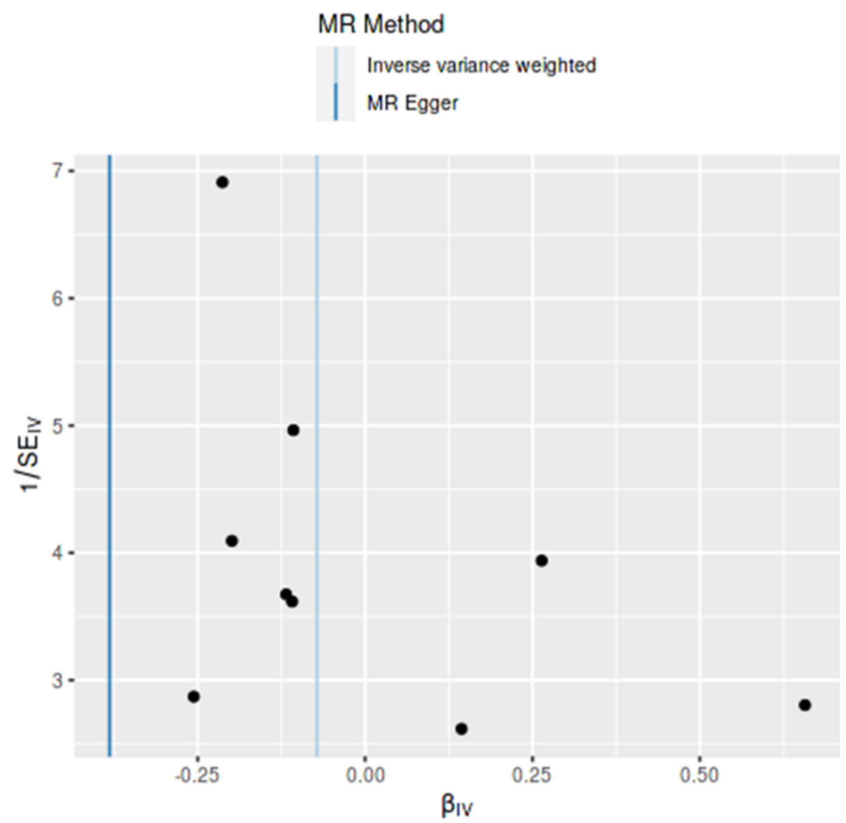

Supplementary Figure S5. Funnel plot of MR estimates of critical COVID-19 and the risk of vascular dementia

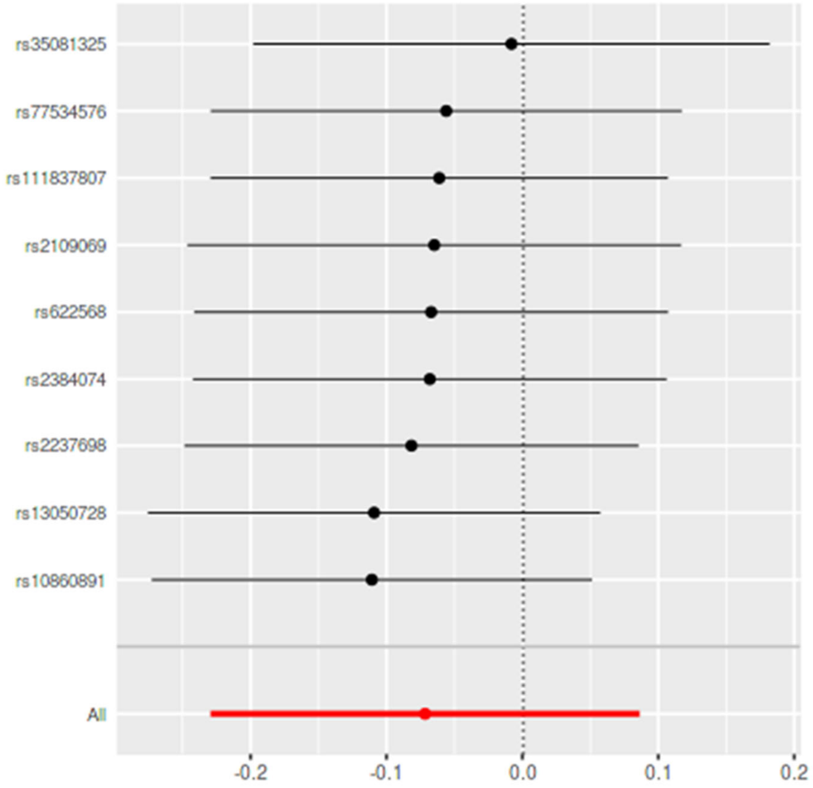

Supplementary Figure S6. Results of leave-one-out sensitivity analysis of critical COVID-19 and the risk of vascular dementia
